# Introduction to the Hologenome Special Series

**DOI:** 10.1128/mBio.00371-16

**Published:** 2016-03-31

**Authors:** Margaret McFall-Ngai

**Affiliations:** Pacific Biosciences Research Center, School of Ocean and Earth Science and Technology, University of Hawaii at Manoa, Honolulu, Hawaii, USA

## EDITORIAL

Because of its strongly theoretical nature, evolutionary biology is a field full of controversy. For example, in the late 1970s, UCLA hosted a forum in which two famous biologists, Motoo Kimura and Francisco Ayala, engaged in a heated debate. Kimura had posited in the late 1960s that molecular evolution is dominated by neutral processes, i.e., genetic drift and random mutation. Ayala argued for the more traditional view, i.e., Darwinian natural selection as the primary force driving evolution of genes and proteins. Ideological camps formed around this controversy, and the debate continues to this day. Nevertheless, these discussions have been a positive driver of research in the field—our view of molecular evolution has benefited and been shaped by the continuing development of the concepts surrounding this debate.

This *mBio* series explores a new topic of controversy in the field of evolutionary biology—the hologenome concept. This view advances the idea that evolutionary forces, both neutrality and selection, act on the host and its symbiotic microbes as a conjoined “holobiont,” and the genomic complement of all partners equals the “hologenome.” The opposing view adheres to the traditional concept that evolution is acting at the level of the individual species that comprise the whole functional biological unit and that this set of closely interacting organisms is most clearly conceptualized and studied as an ecological community. As in the debate between the neutral and selectionist theories, there may be no one answer that applies to all systems under all circumstances, but the exploration of these ideas promises to drive the field forward in exciting ways.

From where does the hologenome controversy arise? Although microbiologists have been strong contributors to the neutralist/selectionist debate of molecular evolution, until recently there has been less focus on macroevolution, i.e., the evolution of separate gene pools, occurring at the species level and above. Through the lens of macroevolutionary concepts, animal and plant biologists had recognized patterns of relatedness among organisms, from species to the higher taxonomic levels, and gained insight into how they arose and diversified across geologic time. It was the pioneering work of Carl Woese, beginning in the late 1970s, that brought macroevolutionary approaches to microbiology. Using the sequences of slowly evolving genes to produce molecular phylogenies, Woese could characterize the relationships of organisms across the *entire* biosphere. The application of molecular features to study microbial evolution, particularly in the last 10 years, continues to have profound effects on all of evolutionary biology. For example, the Modern Synthesis, developed in the 1930s and ’40s, is the most widely held view of evolutionary biology; it couples Darwin’s theory of natural selection with Mendel’s genetics and provides a mechanistic explanation for evolutionary processes in animals and plants. However, because most microbes can acquire genes in a Lamarckian fashion, by lateral gene transfer, much of the biosphere does not adhere to the precepts of the Modern Synthesis. Thus, Eugene Koonin has suggested that we need a “postmodern synthesis” to capture mechanisms of evolution beyond the strictly vertical inheritance of traits. Relevant here is that genome sequencing of the microbiota has demonstrated tight relationships between hosts and their microbial partners. Such relationships span from mitochondria through obligate, vertically transmitted intracellular symbionts, to the extracellular consortia of gut microbes acquired each generation by animal hosts. The host and its microbial partners constitute a complex amalgamation of genomes that is driven by both Darwinian and Lamarckian processes. The stability, connectivity and predictability of symbiotic associations have precipitated a reconsideration of the most fundamental principles of macroevolution. Not only classically trained evolutionary biologists but also others in the field of symbiosis are entering the debate around the hologenome concept.

As the contributions in this series illustrate, evolutionary biology joins a growing set of subdisciplines of biology that have been revolutionized by an emerging realization: that the microbial world is fundamental to all processes of the biosphere across the hierarchy of life, from ecology to cell and molecular biology. For example, with phylo- and metagenomics enabling characterization of the forms and functions of microbial communities, for the last two decades the field of ecology has debated the extent to which the well-developed concepts describing the ecology of macroorganisms can be applied to microbial ecology. Similarly, at the cellular and molecular levels, fields such as immunology, developmental biology, and neuroscience have been transformed by the increasing awareness of our own dependence on the microbial world for growth and health. Evolutionary biology is similarly experiencing the need to question and affirm existing ideas as the field integrates new microbiology-inspired visions. One such vision is the hologenome concept.

When patterns do not fit an existing construct, controversy arises, and … that’s when we learn. The reader would be advised to go into this *mBio* series with an open mind: consider the arguments and embrace the controversy, and think about how such debate will shape our concepts of the biological world. As with the neutralism/selectionist debate, it is likely that the hologenome concept will be a focus of contention for many years to come. Right now, for those of us who are not evolutionary biologists, it is enough to recognize that the very foundations of biology are being shaken by both the integration of microbiology into concepts of macroevolution and the recognition that host-microbe symbioses are a major theme in biological systems.

